# Development and Validation of a Gyroscope-Based Turn Detection Algorithm for Alpine Skiing in the Field

**DOI:** 10.3389/fspor.2019.00018

**Published:** 2019-09-06

**Authors:** Aaron Martínez, Richard Brunauer, Verena Venek, Cory Snyder, Rüdiger Jahnel, Michael Buchecker, Christoph Thorwartl, Thomas Stöggl

**Affiliations:** ^1^Department of Sport and Exercise Science, University of Salzburg, Salzburg, Austria; ^2^Salzburg Research Forschungsgesellschaft m.b.H., Salzburg, Austria

**Keywords:** carving, drifted turns, IMU, sensor, ski

## Abstract

Several methodologies have been proposed to determine turn switches in alpine skiing. A recent study using inertial measurement units (IMU) was able to accurately detect turn switch points in controlled lab conditions. However, this method has yet to be validated during actual skiing in the field. The aim of this study was to further develop and validate this methodology to accurately detect turns in the field, where factors such as slope conditions, velocity, turn length, and turn style can influence the recorded data. A secondary aim was to identify runs. Different turn styles were performed (carving long, short, drifted, and snowplow turns) and the performance of the turn detection algorithm was assessed using the ratio, precision, and recall. Short carved turns showed values of 0.996 and 0.996, carving long 1.007 and 0.993, drifted 0.833 and 1.000 and snowplow 0.538 and 0.839 for ratio and precision, respectively. The results indicated that the improved system was valid and accurate for detecting runs and carved turns. However, for drifted turns, while all the turns detected were real, some real turns were missing. Further development needs to be done to include snowplow skiing.

## Introduction

Turn detection in alpine skiing has been a topic of growing interest over the last 15 years. Turns are the basic unit required for detailed analysis or interpretation of skiing data such as edge angle, symmetry, or turn phases (Müller and Schwameder, 2003; Spörri et al., 2012; Supej et al., 2013; Hébert-Losier et al., 2014). Thus, it is essential to determine when each turn begins (Spörri et al., 2012). Several sensor configurations and methodologies have been proposed to define turn switches. For example, the crossing point between the center of mass (CoM) and the ski trajectories (Supej et al., 2003), the minimum ground reaction force (Nakazato et al., 2011), the instant when the vertical distance of the right ankle joint and left ankle joint to CoM vectors are equal (Fasel et al., 2016b) and the deflection point of the CoM trajectory (Gilgien et al., 2018). Although those methodologies are useful for turn detection, they present some disadvantages that make them not feasible for use on a regular basis. These methods require time consuming preparation, labor-intensive post processing, or alterations in the athlete comfort that make them disadvantageous for regular in-field use.

Inertial measurement unit (IMU) sensor technology has been proposed as a promising alternative approach (Gouwanda and Senanayake, 2008; Fasel et al., 2016b; Yu et al., 2016; Martínez et al., 2019). Smartphones are already used to collect, receive, store, and analyze data collected by wireless sensors, which have progressively become smaller and longer lasting (Kranz et al., 2013; Bondaronek et al., 2018). Based on this development, inquiries are no longer limited to single or double turns, a limitation of some methodologies (Supej et al., 2003; Spörri et al., 2012). Currently, it is possible to perform data collection over longer runs, implementing IMU technologies into the skier's equipment.

Recently, there have been some studies proposing turn detection systems based on simple IMU configurations. Those methodologies are unobtrusive for the athlete avoiding possible alterations in the data related to comfort or technique modification. Yu et al. (2016) placed several IMUs on a single elite skier during part of routine on-slope training for giant slalom and 48 turns were assessed. They computed the angle of each IMU roll axis relative to the vertical to count turns and concluded that the best placements for the IMUs were the pelvis, shank, and foot. Although the system was able to count turns properly, they did not report the turn switch points. A second method was developed with the goal of counting and differentiating right and left turns. Jones et al. (2016) proposed a machine learning approach. They placed a customized sensor consisting on an accelerometer and a gyroscope below the skier's knee. After training the system, ~87% of the turns were detected. Similar to the previous method, this approach aimed only to count turns, not define turn switch point. Both methods were only developed and tested on single skiers. Another methodology was proposed by our research group (Martínez et al., 2019) and was based on two IMUs, placed on the upper cuff of each ski boot. The study was performed in a laboratory environment using a ski-ergometer. The algorithm developed was based on the gyroscope signal and detected turn switch points with a precision of ± 0.03 s. It was hypothesized that this methodology would work for all parallel skiing styles (i.e., carving and drifted turns in all radii), while previous methodologies had focused mostly on slalom or giant slalom race conditions (Supej et al., 2003; Ulrich et al., 2015; Yu et al., 2016). However, this method has not been validated during actual skiing in the field [with the exception of the reported one participant pilot (Martínez et al., 2019)].

Methods such as the aforementioned, allow for extended data collection. As the amount of data collected grows, it seems appropriate to differentiate not only between turns but also between runs since skiing usually consists on multiple sequences of turns. This would allow for a better organization and classification of the data, and potentially for real time analysis. To our knowledge, there is a lack of methodologies to automatically segment ski runs based on non-obtrusive methodology.

The validation of the algorithm used in this study was based on the accurate detection of each turn. Hence, the goals of this study were 2-fold, (1) to further develop and validate the algorithm of Martínez et al. (2019) in field conditions including different turn styles and lengths, and (2) to automatically detect and segment turn sequences.

## Materials and Methods

For the validation and development of the turn detection algorithm, an experiment was designed where an IMU was attached to each ski boot (Martínez et al., 2019) and a camera placed on the chest to serve as a reference. The turn styles assessed were: carving long, carving short, drifted turns and snowplow steering. Participants were instructed to perform long turns over an 8 m corridor, approximately defined by the width of two snow groomer tracks, and short turns within a 4 m corridor, approximately defined by one groomer width. This provided a consistent reference for turn size, without explicitly controlling or defining turn size or shape. Each participant performed a turn sequence including at least 10 turns for each skiing style. The test slope was a red slope (#1) at Mölltaler Glacier in Flattach, Austria, which is steeper in the upper half and moderate in the lower half. Two skiing styles were performed within each run. The first run included carving short on the upper half and carving long on the lower half; while the second run consisted on drifted turns on the upper half and snowplow steering on the lower half. The algorithm development process consisted of several evaluation—development iterations where weaknesses were evaluated, adjusted, and tested again.

### Participants

Eleven male expert skiers (Mean ± SD: Age 26.2 ± 5.9 year; Height = 179.4 ± 5.9 cm; Mass = 77.0 ± 5.4 kg) volunteered for the study. All participants were expert skiers, with experience as ski racers or ski instructors. Before the measurements were conducted, they were informed in detail about the testing procedure, as well as possible benefits and risks of the investigation prior to signing the consent form approved by the local Ethics Committee (EK-GZ: 11/2018). The experiment was conducted in accordance with the Declaration of Helsinki.

### Instruments

**Figure 1:**
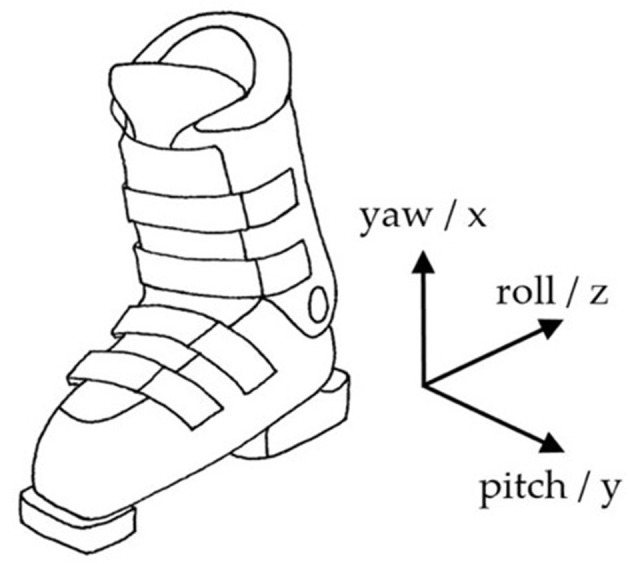
Definition of axes. The positive roll axis is parallel to the ski's surface pointing posteriorly.

An IMU (LSM6DS3, 2.5 × 3 × 0.83 mm, ± 8 g and ± 500 dps full scale, STMicroelectonics, Amsterdam, Netherlands) was placed in the back of the upper cuff of each boot (Hawx 130, Atomic, Altenmarkt, Austria). The X axis of the IMU was aligned with the vertical axis of the boot (yaw) pointing superiorly, the Y with the lateral axis (pitch) pointing to the left, and the Z with the anterior-posterior axis (roll) pointing posteriorly (Figure 1). The IMU was fixed using a tight elastic strap and a customized rigid housing to avoid movement or misalignments. Angular velocities from the gyroscope signals were collected at 833 Hz, analog and digital low-pass filters were applied directly by the IMU after A/D conversion, and the signal was forwarded via Bluetooth at 64 Hz. The sensor data was collected and stored by an in-house smartphone application (SkiSense App, Salzburg Research, Salzburg, Austria).

In order to define reference turn switch points and determine the proper detection of turns (e.g., real turns not detected or turns erroneously detected), all runs were recorded with a camera (Hero4 Session, GoPro, San Mateo, CA, USA) with a sampling rate of 25 Hz. The camera was placed on the skiers' chests using an adjustable chest harness, and positioned such that the field of view included both skis, boots and the surrounding area.

### Data Preparation

**Figure 2:**
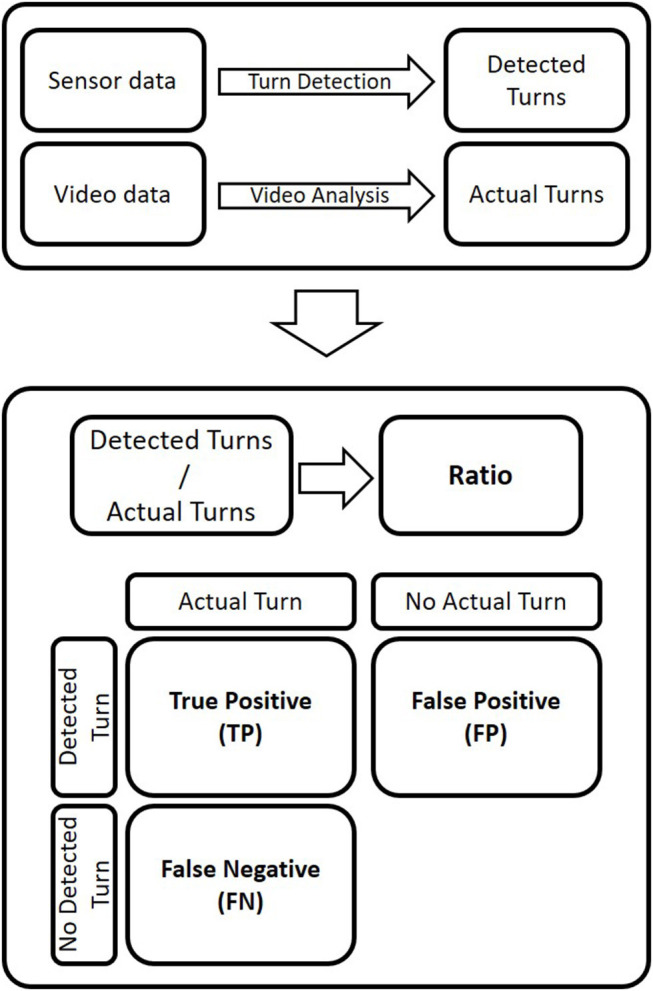
Data flow from field measurements to data used for the validation and performance metrics. Determination of the ratio between the number of turns detected and actual turns and computation of the metrics used to calculate precision and recall.

Video data was analyzed by experienced raters, labeling relevant events or context, such as turn switch point, turn direction, or jump. Turn switch points in the video analysis were defined as the frames where the surfaces of the skis were flat on the ground. Video and sensor data were synchronized using a jump event at the beginning and end of each trial. The jumps were detected in the vertical axis of the accelerometer and synchronized with the frame at landing. For validation, the actual turns obtained by the video analysis and the number of detected turns were used. The detected turns were computed by applying the turn detection algorithm (Martínez et al., 2019) to the sensor data (see Figure 2).

### Evaluation Design

In order to validate the turn detection algorithm, three performance metrics were implemented: (1) ratio, (2) turn count precision, and (3) recall (see below). All steps of evaluation (i.e., data acquisition, video analysis, signal evaluation) were conducted in a blinded procedure.

- *Ratio:* the quotient between the number of turns detected by the algorithm and the number of turns labeled in the video data (see Figure 2). If the ratio is >1, the turn detection algorithm overestimates the number of actual turns, and vice versa. A ratio of one means that the number of detected turns and actual turns are the same, indicating a good performance of the turn detection. However, it does not indicate if all the detected turns were real turns, since only the number of turns is evaluated.
- *Turn count precision (P):* the proportion of turns detected by the algorithm that are actual turns (see Equation 1).
- *Recall (R):* the proportion of actual turns detected by the turn detection algorithm (see Equation 2).

In all metrics a value of 1 means a perfect performance.

To calculate the turn count precision and recall values, the following metric were determined (Figure 2):

- True positives are determined by a detected turn that was an actual turn (based on video). A correct detection is characterized by the correct turn direction (either left or right turn) and by the difference in time between the detected and the actual timestamp. If the time difference is smaller than half the mean duration of actual turns (for a run), it is a true positive.
- False positives reflect the number of detected turns that are not actual turns.
- False negatives are the number of actual turns that are not detected by the turn detection algorithm.

(1) $$\begin{array}{rcl} P\; & = & {TP\;•\;\left(TP\;+\;FP\right)}^{-1} \end{array}$$

(2) $$\begin{array}{rcl} R & = & {TP\;•\;\left(TP+FN\right)}^{-1} \end{array}$$

Where P is the turn count precision, TP the true positives, FP the false positives, R the recall and FN the false negatives.

### Algorithm Development

The purpose of the following algorithm was to determinate turn switches and sequences of parallel skiing turns. To keep the algorithm simple, it was assumed that a turn switch is a single point in time where both skis change from the uphill to downhill set of edges simultaneously. The performance metrics and video recordings were used to evaluate the performance of the current algorithm iteration (see section Evaluation design) and detect weaknesses.

**Figure 3:**
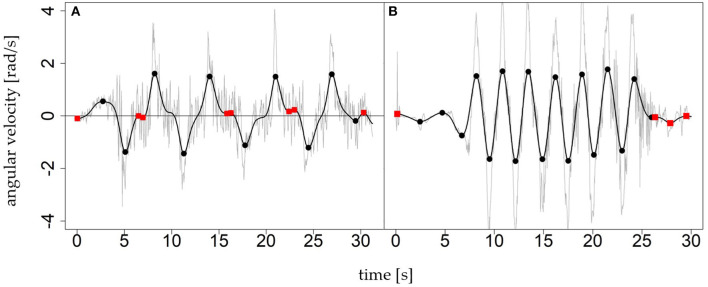
Two examples of the roll signal ω_*roll*_ (gray) and decision signal ω_*ds*_ (black) of ski runs with several carving turns **(A)** and short turns **(B)**. The markers show local extrema; the black circles represent turn switches and the red squares are artifacts to be recognized and discarded.

The starting point was the evaluation of the algorithm developed under laboratory conditions by Martínez et al. (2019). Matlab (R2017B, MathWorks, Natick, MA, USA) and R (3.5.1, R Core Team, Auckland, New Zealand) were used to test and develop the algorithm. The roll axis (z) of the gyroscope signal from the left leg $\omega_{roll}^{l}$ and right leg $\omega_{roll}^{r}$ [rad/s] were used as input signal to calculate the turn switch points (Figure 1). High rotation rates (angular velocity) in the roll axis can have different causes (e.g., turning or skating). To restrict high rates to parallel turns, the arithmetic mean of both sides ω_*roll*_ smooths unilateral rotations. In the next step, a fourth-order zero-lag 0.5 Hz low-pass Butterworth (BW) (Martínez et al., 2019) was applied to produce a decision signal (ω_*ds*_) that describes the rotation rate in the roll axis (Figure 3).

**Figure 4:**
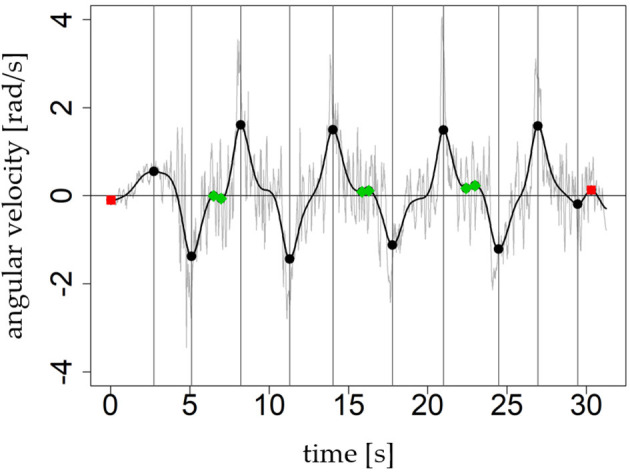
The graph shows the proposed three types of labels for local extrema. Local extrema which are labeled as *switch* are black circles and additionally highlighted by the gray vertical line. The red squares represent local extrema with label *eliminated* and the green rhomboids outlines those with label *noise*. The average turn duration in this example is 2.97s and is longer than in Figure 3.

Figure 3 shows the local extrema in the two exemplary decision signals ω_*ds*_ = *BW*_0.5_(−ω_*roll*_). The left graph shows a sequence of nine carving turns (mean ± SD; turn duration of 2.97 ± 0.43 s), the right graph a sequence of 16 short turns (turn duration 1.04 ± 0.41 s). The minus before ω_*roll*_ flips the positive direction to anterior in agreement with the movement direction. The extrema indicate possible turn switches, whereby the sign of a value in ω_*ds*_ determines the turn direction. As Figure 3 shows, local extrema are not necessarily caused by turn switches (red dots). There are other causes, especially when the signal oscillates with small amplitudes around zero after the skier has stopped. Furthermore, the constant cut-off frequency of 0.5 Hz is a trade off between damping the rotation rate of short turns too much and fostering artifacts (red dots/local extrema within turns) in long turns. Thus, as a next step, the algorithm goes through the sequence of all local extrema and labels each local extrema (Figure 4):

- *Switch:* This kind of local extrema indicates actual turn switches from left to right or right to left.
- *Noise:* Local extrema that happen within a turn are called noise. If a skier performs turns with longer duration (e.g., slowly skidded parallel turns or carving turns with a long stable edge angle), the decision signal ω_*ds*_ shows some artifacts as saddle points or small counter oscillations (Figure 4, green dots). These artifacts result in local extrema which are “within a turn” and do not indicate an actual turn switch.
- *Eliminated:* All other local extrema must be eliminated from the algorithm. These extrema are “outside a turn” and caused by small oscillations when the skier is not turning. For example, waiting, walking, or skiing straight downhill. The eliminated extrema are used to define the beginning and end of turn sequences.

Several heuristic rules were used for the labeling process:

1. *Switch*: Look at each pair of two consecutive local extrema: If two consecutive local maxima have a high rotation rate in *abs*(ω_*ds*_), opposite sign and a time distance >0.3 s and smaller than 5.0 s then label both as *switch*.
2. *Noise*: Look at each sequence of four consecutive local extrema: If both outer extrema fulfill rule 1 and both inner local extrema not and if the rotation rates in *abs*(ω_*ds*_) of the inner are smaller than the outer then label both inner as *noise*.
3. *Eliminated*:
  - Look at each local extrema: If it is not labeled as *switch* or *noise* and if the rotation rate is smaller than a threshold then label as *eliminated*.
  - Look at each local extrema: If it is not labeled as *switch, noise* or *eliminated* and if it has the same sign in ω_*ds*_ as its predecessor then label as *eliminated*.
  - Look at each local extrema: If it is not labeled as *switch, noise*, or *eliminated* apply a decision tree (see below) to label it as *eliminated* or not.
  - Look at each local extrema: If it is not labeled as *switch, noise* or *eliminated* and if the predecessor and successor are *eliminated* then label as *eliminated*.
4. Remaining: Look at each local extrema: If it is not labeled as *switch, noise*, or *eliminated* then label as *switch*.

*Decision tree* (3c): the goal of the machine learning task was to obtain a simple rule that removes all remaining local extrema outside a turn sequence because these local extrema should have the label *eliminated*. If we do not have such a rule, rule 4 cannot be applied. A decision tree was trained by means of machine learning. The intent of the decision tree was to discriminate for each local extrema between the classes *within a turn sequence* or *not-eliminated* and *outside a turn sequence* or *eliminated*. Data were labeled by manually defining the time ranges of the turn sequences, and a cross-validation was used to avoid overfitting the decision tree. Obvious features as $\omega_{roll}^{r}$, $\omega_{roll}^{l}$, ω_*roll*_, ω_*ds*_, ω_*ds*_ (change in angular velocity between two consecutive local extrema), its absolute values *abs*(ω_*ds*_) and *t* were used to search the most expressive features. The result was the decision tree: *abs*(ω_*ds*_) ≤ θ_1_ ∧ *t* ≤ θ_2_ → *eliminated*. That means that the absolute value ω_*ds*_ and the *t* (time between two consecutive local extrema) were identified by the machine learning task as most expressive. The variables θ_1_ and θ_2_ are the modeling parameters of the machine learning model thresholds.

**Figure 5:**
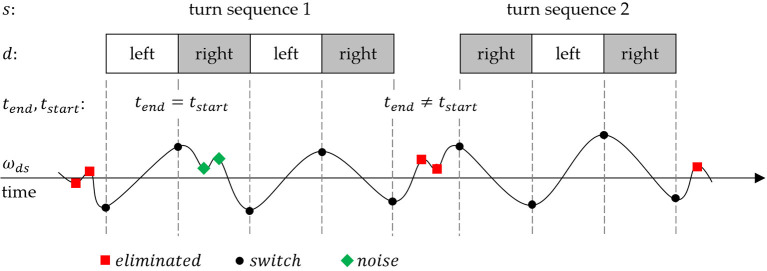
The lower part shows an exemplary decision signal ω_*ds*_ with local extrema of type switch, noise and eliminated. The corresponding derived turns and turn sequences are shown above.

The result of the previous step is a turn sequence (TS) of labeled local extrema *TS*_*ds,n*_((_*t*_*n*_, ω_*ds*_(*t*_*n*_), *l*_*n*_))*n*∈ℕ_. Next, the algorithm derives the turns (*t*_*start*_, *t*_*end*_, *d, s*) from the sequence *TS*_*ds,n*_. The timestamps *t*_*start*_ and *t*_*end*_ are the start and the end of a turn (turn switch points), the value *d* ∈ {*left, right*} indicates turn direction and *s*∈ ℕ is the number of the turn sequence to which the turn belongs (Figure 5). The value of *d* depends on the orientation of positive roll axis. Figure 1 shows a right-handed coordinate system with positive roll axis in the posterior direction. Thus, local maxima (positive rotation) indicate turn switches from left to right and local minima (negative rotation) from right to left. A continuous alternating sequence of turns is given, if *t*_*end,i*−1_ = *t*_*start,i*_ respectively if the end of turn *i* − 1 is the start of turn *i*, then *s*_*i*_ = *s*_*i*−1_. If *t*_*end,i*−1_≠*t*_*start,i*_, then turn *i* starts a new turn sequence and *s*_*i*_ = *s*_*i*−1_ + 1 (Figure 5). The start value of *s*_1_ is 1. To determine the sequence of turns *T*_*ds,m*_((_*t*_*start,m*_, *t*_*end,m*_, *d*_*m*_, *s*_*m*_))*m*∈ℕ_ (*m* < *n*), the algorithm iterates through the sequence and merges consecutive extrema with label switch to a turn if and only if they are no interruptions from local extrema with label eliminated. This rule produces a continuous turn sequence with *t*_*end,i*−1_ = *t*_*start,i*_. Local extrema with label noise (per definition “within turn”) were ignored because they do not interrupt a turn. Eliminated local extrema (per definition “outside turn”) indicate an interruption of a consecutive turn sequence and causes *t*_*end,i*−1_ ≠ *t*_*start,i*_ and *s*_*i*_ = *s*_*i*−1_ + 1. The previous heuristic rules ensure that in a turn sequence each switch is followed by a switch with opposite sign, even in case were local extrema with label noise are between. A strict alternation of the sign is necessary because otherwise there is no alternation of left and right turns, which is not obvious.

**Figure 6:**
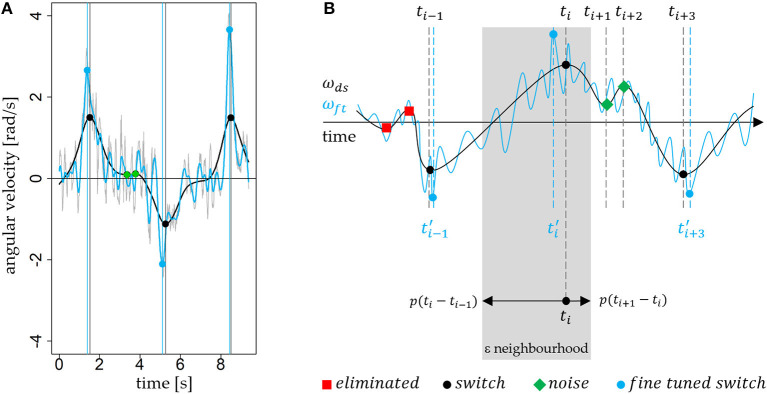
**(A)** Comparison of the raw signal −ω_*roll*_ (gray), the filtered decision signal ω_*ds*_ (black, 0.5 Hz) and fine tuning signal ω_*ft*_ (blue, 3.0 Hz). The vertical blue and black lines show the effect of fine tuning, and the small shift in time. **(B)** The graph outlines (in gray) the definition of ε neighborhood graphically. A *p* of 0.6 defines a ε neighborhood (gray) where 60% of the distance to predecessor and successor extremum are part.

The decision signal is a strongly smoothed signal. The rotation rates represent the general rotation behavior of (parallel) turning during skiing. The degree of smoothing between raw and decision signal can be observed in Figures 4, 6A. To determine the turn switches more accurately, a higher cut-off frequency seems to be more advisable. As shown in the study of Martínez et al. (2019), *f*_*c*_ = 3.0 Hz is a proper cut-off frequency to perform such a “fine tuning.” Therefore, a new fine tuning signal is defined by ω_*ft*_ = *BW*_3.0_(−ω_*roll*_) (Figure 6A). Then the algorithm searches for each (*t*_*i*_, ω_*ds*_(*t*_*i*_), *l*_*i*_) the global maximum or minimum in ω_*ft*_ in a time window (an asymmetric ε neighborhood) around the turn switch timestamp *t*_*i*_. The algorithm “fine tunes” only local extrema labeled as switch and defines the global maximum or minimum $\omega_{ft}(t_{i}^{\prime })$ at $t_{i}^{\prime }$ as the new “fine tuned” turn switch. A percentage value or factor *p* ∈ [0, 1] defines the size of the asymmetric ε neighborhood (Figure 6B): [*t*_*i*_ − *p*(*t*_*i*_ − *t*_*i*−1_), *t*_*i*_ + *p*(*t*_*i*+1_ − *t*_*i*_)].

For the succeeding evaluation we use 60% for the ε neighborhood, thus *p* = 0.6. Finally, the fine tuned turn switches, turns and turn sequences are based on the sequence $TS_{ft,n}{\left(({t^{\prime }}_{n},\omega_{ft}({t^{\prime }}_{n}),l_{n})\right)}_{n\in \mathbb{N}}.$. Finally, the new sequence of fine tuned turns $T_{ft,m}\;{\left(({t^{\prime }}_{start,m},{t^{\prime }}_{end,m},d_{m},s_{m})\right)}_{m\in \mathbb{N}}$ is given by substituting all *t* in *T*_*ds,m*_ with *t*′.

## Results

The results of the turn detection evaluation for each skiing style (short and long carved, drifted, and snowplow turns) are shown in Table 1. The ratio between the detected and the actual carved turns (long and short turns pooled together) was 0.997, the best value of the different skiing styles analyzed (drifted 0.833, snowplow 0.538). A ratio of 0.997 indicates that the number of actual carving turns was slightly underestimated by the turn detection algorithm.

**Table 1:** Evaluation metrics for each skiing style and group of styles in number of turns; ratio, turn count precision, and recall of each adapted confusion matrix.

**Turn Sequence**	**AT**	**TP**	**FP**	**FN**	**Ratio**	**Precision**	**Recall**
Short carved turns	231	230	1	2	0.996	0.996	0.991
Long carved turns	143	143	1	0	1.007	0.993	1
Drifted turns	132	109	0	22	0.833	1	0.833
Snowplow	104	47	9	57	0.538	0.839	0.452
**TURN SEQUENCE GROUPED**							
Carved	374	373	2	2	0.997	0.995	0.995
Parallel	506	482	2	24	0.953	0.996	0.953
All	610	529	11	81	0.867	0.980	0.867

*AT, actual turns; TP, true positives; FP, false positives; FN, false negatives; Precision, turn count precision. Carved includes short and long carved turns. Parallel includes carved and drifted turns. All includes parallel and snowplow*.

Investigating the turn precision measures of the algorithm for carved turns, almost all detected turns were correctly detected (turn precision was higher than 0.993 for both turn sequences). The recall measures were also high with values >0.990, which indicates that almost all actual turns were detected by the algorithm.

## Discussion

The purpose of this study was to develop and validate a turn detection algorithm for parallel alpine skiing turns in the field. The assumption that a turn switch is a single point in time where the edge changes simultaneously for both skis is not too critical if the turns have seamless transitions, and the skier performs parallel styles. Conversely, the algorithm is not able to count all turn styles accurately. For example, snowplow and drifted turns with low dynamics or straight piste crossings cause problems. However, the proposed methodology was aimed at continuous turn sequences of parallel style turns, as carved turns are usually the main subject of interest.

**Figure 7:**
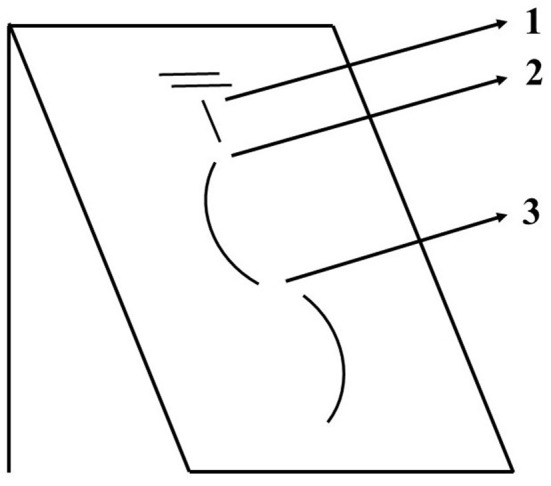
Schematic representation of the beginning of a turn sequence and three different options for the first detected turn. Option 2 was the desired outcome.

For validation, tests were performed by several subjects with diverse techniques, turn lengths, and snow conditions. The instructions for the different turn lengths aimed to define different types of turns (e.g., short and long) while allowing the participants to perform slightly different turns. We believe that not having an exact turn length adds an extra variability that is beneficial to develop a more robust algorithm. The natural variation in snow conditions across the 4 days of testing provided an additional layer of variability, adding to the robustness of the algorithm. The results of 374 carved turns (0.997 ratio, 0.995 precision, and 0.995 recall) prove the validity and robustness of this turn detection algorithm for this particular turning technique. Misdetections during carved turns were only observed at the beginning of the turn sequences; however, they were mostly counted correctly. The three different options observed for the initial turn detected in a turn sequence are shown in Figure 7. From 32 parallel turn sequences analyzed in this study, five were not properly detected. The system aimed to detect the first edge change out of the fall line (option 2 in Figure 7) and this first turn switch of each run was ignored and not included. Out of the five turn sequences not properly detected, two counted an extra turn (FP, option 1 in Figure 7) and three failed to count one turn (FN option 3 in Figure 7). Besides this issue with the beginning of the turn sequences, for carved turns, there were no other misdetections, all the turns within sequences were properly detected.

Ratio and recall for drifted turns (0.833 both values) were lower than for carved turns. The turn detection algorithm worked properly for all participants but four. For those participants, the missing turns were not isolated but consecutive (e.g., 2, 4, 6, and 9 consecutive turns). All those turns had some common characteristics. They were longer slow turns, which produced a less dynamic movement, and consequently were more affected by the snow conditions, in those cases, bumpy surfaces. The algorithm is prepared to recognize and cancel some noise (see Figure 5); however, when there is too much noise (e.g., more than two extra local maxima are detected between turns) the turn sequence is finalized. If there are several turns under those conditions, it does not report multiple sequences with single turns, but rather discards most of the turns since the first turn of every sequence is discarded. The possible misdetection of the first turn and the long low dynamic turns were the causes of some misdetection of turn sequences. When turn detection worked accurately, the sequences were properly defined and cut. Discriminating the signal depending on how dynamic the movement is could help to further develop the algorithm and overcome those missing turns.

Although the algorithm was developed for parallel turns, the performance during snowplow steering was also evaluated. While the turn count precision was acceptable, ratio and recall were unacceptably low (0.839, 0.538, and 0.452, respectively). As expected, most of the detected turns were actual turns, but many real turns were not detected. The snowplow turning style is completely different than drifted or carved turns. The legs do not have synchronized rotation rates in roll axis. The main movement does not rotate around the anterior-posterior axis, but around the vertical axis. Consequently, an algorithm that only takes into consideration the anterior-posterior axis might not be the most effective approach. However, snowplow turns are usually turns during learning and they are not used very often in everyday skiing. Thus, the focus on parallel turns is obvious, at least from a practical perspective. Further development, probably including a combination of more than one axis or a turn style detection system, would be needed for the automatic detection of snowplow turns.

Over the different iterations of the development process, modifications were included in the algorithm. These modifications were needed in order to cope with signal features that differed between in-lab (Martínez et al., 2019) and real skiing conditions. Due to the variable snow conditions, styles, turn lengths, and skiers, the signals recorded during skiing had more variability and some recurrent differences when compared to simulated turns on a ski glider. To confront this issue, some recurrent misdetections that did not corresponded with turn switch points (e.g., noise within turns or artifacts after stopping) were corrected, adding new rules to the algorithm and thus making it less simple. One of the additions was the decision tree. This decision tree is easy to implement by a single if condition. Thus, we preferred to use just a simple machine learning model, which is still human interpretable and implementable, to provide an additional heuristic rule. Such a simpler problem statement needs fewer data. However, it would be possible to generate a decision tree that is able to replace all the rules from 1 to 4. Such a decision tree would need more labeled data and the input for learning data would have to be more complex. It would be necessary to analyze at least a short sequence of local extrema. In our case, we looked only on the local extremum and its predecessor. This could be an approach for further optimization and automatization. However, for our proposed algorithm, machine learning was applied to provide final heuristic rule for the remaining local extrema. Furthermore, we limited the use of machine learning approaches in order to keep computational cost low, as this algorithm could be implemented in a smartphone app.

During the development process, some algorithm rules, and parameters were manually optimized to fine tune the algorithm. However, the algorithm presented in this publication is fully automatic, and does not require manual input. The results could however be influenced by IMU specifications such as sensitivity, sampling rate, and orientation.

For the validation of the turn detection algorithm, events such as falls were excluded from the study. One of the subjects fell during one trial, and two turns were detected based on this fall. Thus, in case of a fall, the algorithm might detect some extra turns.

The present methodology presents some advantages when compared to previously proposed methods. Yu et al. (2016) successfully counted turns using a single IMU. However, they only assessed one participant during a single giant slalom race trial, and the method was based on the angle with respect to the vertical. To properly calculate the orientation of the sensor, integration and drift correction computation procedures are needed (Seel et al., 2014; Fasel et al., 2018). This type of computation usually requires post-processing the data and makes it more difficult to perform analysis in real time and potentially limits the applications of this method. It is also important to consider that their goal was to count turns, therefore they did not report the turn switch points. The limitations of using video recording during skiing are well-known. There is limited capture volume (Supej et al., 2003; Fasel et al., 2016a), and it requires time consuming preparation and post processing (Reid, 2010). Furthermore, recent work from our research group showed that even in ideal lab conditions, the determination of the turn switch point using video recordings as a reference is challenging. Different raters, even with the same instructions, select different frames as the turn switch point (the reported range between raters was 51.4 ms; Martínez et al., 2019). For this reason, precision in the field was not measured in this study, but we can reasonably assume similar precision to that observed in the lab (± 0.03 s). Since the nature of the movement on the ski-ergometer and during real skiing both follow our model of pendular movement, the basic features identified as turn switch points within the signals should be similar on the ski-ergometer and in the field. As such, the turn switch point determined by video analysis was only used as a reference to determine if the measured turn switch point corresponded to an actual turn switch.

## Conclusion

In conclusion, the developed turn detection system is a valid, robust, and generally simple tool to detect parallel turns (carved and drifted) in alpine skiing with an accuracy of 95.3%. For carving turns, the algorithm is able to accurately detect 99.7% of actual carving turns. The algorithm is based only on a single axis of gyroscope data. Simple IMUs, securely mounted on both ski boots, provide sufficient data for the segmentation of time series signals into both turn sequences and single turns. Both are very convenient data structures for further analysis tasks. The approach is applicable for in-field studies because the devices do not disturb skiing and the algorithm is fully automated. Further research is needed to assess the performance of the system for skiers with different skill levels, or to include the detection of other turn styles, such as snowplow turns.

## Data Availability

The datasets generated for this study are available on request to the corresponding author.

## Ethics Statement

The studies involving human participants were reviewed and approved by Ethics Committee of the University of Salzburg. The participants provided their written informed consent to participate in this study.

## Author Contributions

TS, RJ, and RB: conceptualization and investigation. TS, RJ, RB, MB, and AM: methodology. RB and MB: software. AM, VV, CT, and CS: validation. RB, MB, VV, and AM: formal analysis. TS and RB: resources. RJ, RB, and VV: data curation. AM, RB, and VV: writing-original draft preparation and visualization. CS, RJ, MB, TS, VV, and RB: writing-review and editing. TS: supervision, project administration, and funding acquisition.

### Conflict of Interest Statement

The authors declare that the research was conducted in the absence of any commercial or financial relationships that could be construed as a potential conflict of interest.
